# Evaluation of dosimetric characteristics of a ternary nanocomposite based on High Density Polyethylene/Bismuth Oxide/Graphene Oxide for gamma-rays

**DOI:** 10.1038/s41598-022-23605-y

**Published:** 2022-11-05

**Authors:** Amir Veiskarami, Dariush Sardari, Shahryar Malekie, Farshid Babapour Mofrad, Sedigheh Kashian

**Affiliations:** 1 Department of Medical Radiation Engineering, Science and Research Branch, Islamic Azad University, Tehran, Iran; 2 Radiation Application Research School, Nuclear Science and Technology Research Institute, P.O. Box 31485-498, Karaj, Iran

**Keywords:** Sensors and biosensors, Experimental nuclear physics

## Abstract

This research aims to investigate a ternary nanocomposite based on High Density Polyethylene/ Bismuth Oxide/Graphene Oxide (HDPE/Bi_2_O_3_/GO) at various concentrations. Solution method was used to fabricate the samples. FESEM-EDX mapping, AFM, TEM, XRD, XPS, FTIR, and TGA/DTG analyses were carried out on the samples. XRD analysis demonstrated a semi-crystalline behavior for the samples. TEM analysis exhibited a cauliflower-like structure of the material. The sample was irradiated by gamma-rays of ^60^Co source over the dose rate of 30–254 mGy/min and the electric current was measured as the response of the real-time dosimeter. Thus, various dosimetric characteristics were performed, namely linearity, angular dependence, energy dependence, bias-polarity, field size, and repeatability of the data. Results showed that response of the dosimeter was linear in the range of the investigated dose rate. The sensitivity of the 60 wt% Bi_2_O_3_ sample was measured as 3.4 nC·mGy^−1^. The angular response variation was 20% for normal beam incidence. The response of the dosimeter to assess the energy dependency was obtained as 2.2% at the radiation field of the ^137^Cs and ^60^Co beams. The dosimeter response was dependent on the bias-polarity, with maximum discrepancy of 11.1%. The dosimetry response was highly dependent upon the radiation field size. The repeatability of the dosimeter response was measured with standard deviation less than 1%. As well, the dosimeter response during the one-hour irradiation was stable with a standard deviation of 0.66%. Results showed that considering some correction factors, this material can be used for dosimetry of gamma-rays at the therapy level.

## Introduction

Recently, polymer-nanocomposites have attracted significant attention from scientists for use as radiation protection, sensors, detectors, and dosimeters^1–10^. These materials have many advantages due to their lightness, flexibility, ease of processing, being tissue equivalent, and relatively low cost.

The selection of filler and matrix materials in a polymer-nanocomposite improves the performance of a particular system, depending on its type of operation. Graphene as a promising material exhibits extensive properties of mechanical, optical, and electrical with huge potential applications in the industry^11^. One of the most important determining factors in improving the performance of detection and dosimetry systems is increasing their sensitivity. In fact, by adding fillers with a high atomic number such as bismuth oxide (Bi_2_O_3_, Z = 83 for Bi) to a polymer matrix, the sensitivity of the nanocomposite can be improved to some extent. If the filler particles are homogeneously distributed in the polymer matrix, it is expected that the sensitivity and accuracy of the detector/dosimeter system will increase significantly. Therefore, the use of Bi_2_O_3_ nanoparticles can significantly affect the response of the detector/dosimeter due to the increase in the photon absorption cross-section.

Irradiation of a polymer nanocomposite with gamma-rays and high-energy photons through interactions such as photoelectric effect, Compton scattering and pair production leads to the production of electric charges (electron–hole) in the sensitive volume of the material. By applying a suitable electric field to the material, electric charges can be collected and converted into signals. The results of some studies have shown that irradiation can lead to charge trapping and recombination in the material^12,13^. This problem can be solved by adding a tiny amount of graphene oxide (GO) with excellent electrical conductivity to a polymer matrix. The main advantage of using GO in this work is that the OH edge functional groups establish a covalent bond with the polymer chains and will have better compatibility with the polymer matrix^14^. Dosimetry responses of polymer/carbon nanostructures have been investigated for gamma-rays in the previous studies by this research group^6,9,15,16^. For polymer/carbon nanostructures, the amount of carbon additives can affect the detector/dosimeter response consequently^17,18^. Adding carbon nanostructured materials such as GO and carbon nanotubes (CNTs) into a polymer matrix in a particular weight fraction entitled electrical percolation threshold (EPT), leads to a subsequent sudden increase in electrical conductivity of the nanocomposite of several orders of magnitude^4,19,20^. On the other hand, the weight percentage of GO in the nanocomposite should not exceed the EPT value, because the amount of dark current in detection/dosimetry systems increases and reduces the sensitivity. Thus, the EPT value is an essential parameter to be used in the detectors or dosimeters based on carbon nanostructures^5^. It is worth pointing out here that the EPT for polymer/carbon nanostructures was calculated or measured between 0.12 and 2.2 wt%^21,22^.

Polymer nanocomposites show low sensitivity to gamma-rays due to their low photon absorption cross-section. Thus, to overcome this problem, Bi_2_O_3_ nanoparticles with a high atomic number are added to the polymer matrix. Bi_2_O_3_ nanoparticles, due to having a high photon absorption cross-section and thus increasing the probability of photoelectric phenomenon in the interaction of photons with matter, can increase the sensitivity of the nanocomposites for detection and dosimetry purposes. Intaniwet et al. investigated the effect of adding heavy metal oxide nanoparticles in a semiconducting polymer to enhance the sensitivity of the detectors for 17.5 keV x-rays^8^. The gamma-ray dose rate was measured in real time by applying an electric field of 1–5 kV/mm using pure PMMA and LDPE polymers^23,24^.

The dispersion state of the nanoparticles into a polymer matrix is a challenging issue due to the agglomeration effect of the inclusions, especially at higher volume fractions^25^. In this exploration, HDPE with a repeat unit of –[C_2_H_4_]– as a thermoplastic polymer exhibiting a semi-crystalline structure was selected as a polymer matrix. Also, a high-Z material, namely Bi_2_O_3_ nano-powder (Z = 83 for Bi) with a density of 8.9 g/cm^3^ was chosen as the filler. The high surface to volume ratio of the Bi_2_O_3_ nanoparticles causes improvement in the photon absorption cross-section of the polymer nanocomposites in specified energies.

In this research, a novel ternary real-time dosimeter at the therapy level based on the HDPE/Bi_2_O_3_/GO nanocomposite was fabricated. Then, various dosimetry characteristics, including the dependence of dosimetry response to linearity, angular, energy, bias-polarity, field size, and repeatability of the measurements were investigated for the nanocomposite containing 60 wt% Bi_2_O_3_ and 0.1 wt% GO under the gamma-rays at the Secondary Standard Dosimetry Laboratory (SSDL) of Karaj-Iran.

## Material and methods

### Samples preparation

**Figure 1:**
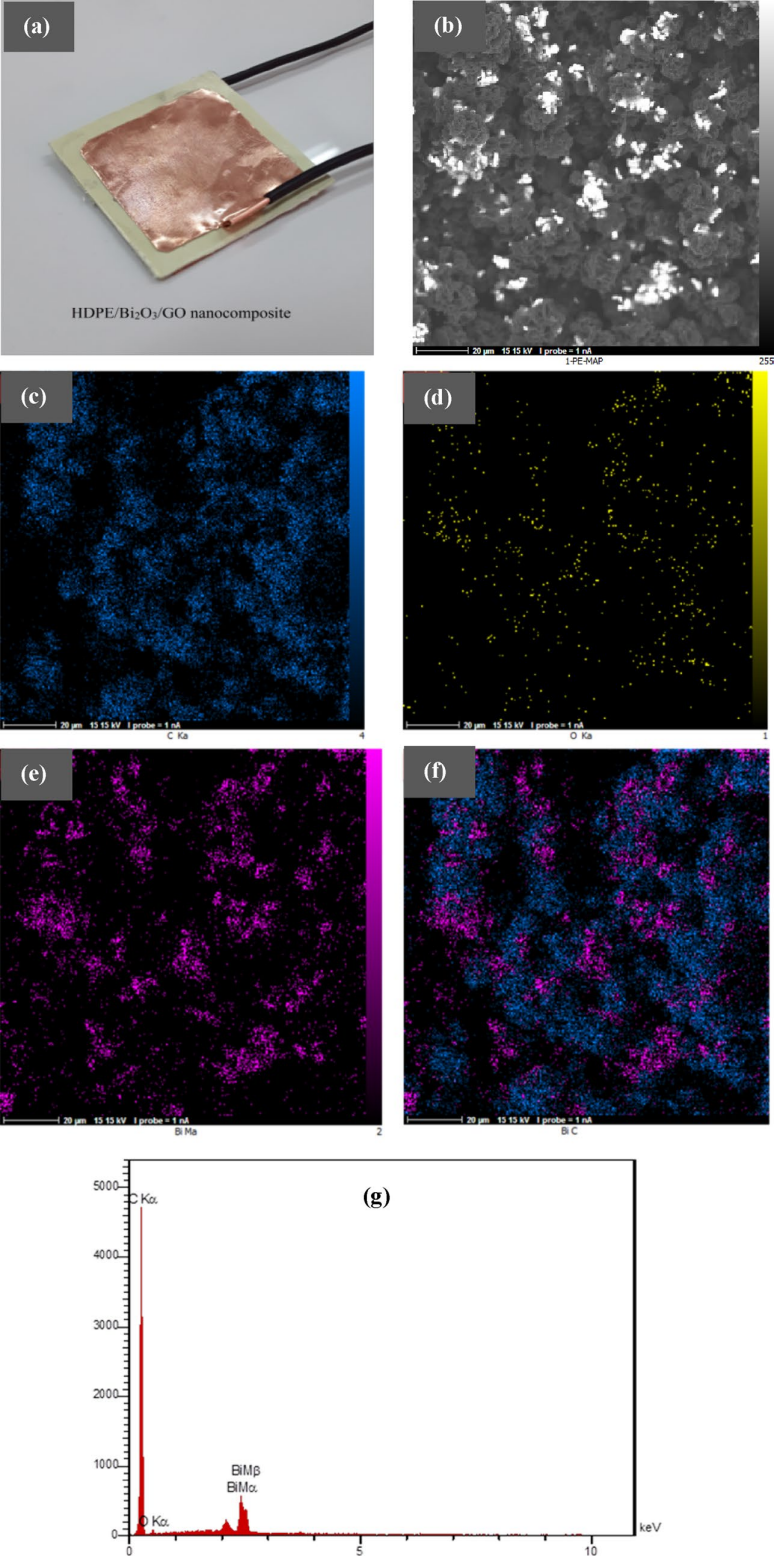
(**a**) A view of the fabricated dosimeter with copper electrodes, (**b**) FFESEM–Mapping of the B_60_ sample exhibiting (**c**) Carbon element (dark blue), (**d**) Oxygen element (yellow), (**e**) Bismuth element (Purple), (**f**) overlay of Carbon, and Bismuth elements, and (**g**) EDX spectrum of the B_60_ sample.

In this experimental work, HDPE granules with density of 0.93 g/cm^3^ were supplied from the Iranian-Khuzestan Petrochemical Company. Bi_2_O_3_ nanopowder with a density of 8.9 g/cm^3^ and an average particle size of 90–210 nm was prepared from Sigma-Aldrich. The GO nanopowder with 6–10 layers and a total thickness of 3.4–7 nm was obtained from the US Nano Inc. P-Xylene (para-Xylene) was provided as a chemical solvent for HDPE. A hot plate magnet stirrer was used to dissolve HDPE in p-xylene solvent at 90 °C. Also, to achieve uniform dispersion of the Bi_2_O_3_ nanoparticles in HDPE matrix, an ultrasonic probe (Hielscher-UP400St) was used for 1 h. Finally, HDPE/Bi_2_O_3_/GO nanocomposite containing 60 wt% Bi_2_O_3_ and 0.1 wt% GO was fabricated using a hot press at the same dimensions of 4 × 4 cm^2^ with constant thicknesses of 1 mm. In the molding stage, first pre-heating was performed for 10 min, and then it was subjected to hot pressing for 10 min at 190 °C. After fabricating the sample (Fig. 1a), to fabricate the electrodes on two surfaces of the samples, the copper sheets with a thickness of 100 µm were connected to the surfaces of the samples using the silver paste (Supplementary Note 1). In Table 1, the details of each sample are described. To investigate the effect of Bi_2_O_3_ nano-fillers on the dosimetry response, four samples were fabricated in different concentrations of 0, 20, 40 and 60 wt% (Supplementary Note 2, Fig. 1E). Also, to explore the effect of GO nano-fillers on the dosimetry response, several samples were constructed in different GO wt% namely 0, 0.1, 0.5, 1 and 2 wt% (Supplementary Note 3, Fig. 3E).

**Table 1:** The nano-fillers weight percentage (wt %) for fabricating the samples.

Sample ID	Polymer matrix	Bi_2_O_3_ wt %	GO wt %
B_0_	HDPE	0	0
B_60_	HDPE	60	0.1

### Instruments

To irradiate the samples, gamma irradiators, namely Picker-V9 and Theratron-780, and OB-85 available at the Secondary Standard Dosimetry Laboratory (SSDL) Karaj-Iran, were used at different source-surface distances (SSDs). Also, an electrometer model Supermax Standard Imaging was employed to measure the electric charge during the irradiation at fixed time steps of 15 s. In the all measurements, a low-noise cable was used to connect the nanocomposite dosimeter to the Supermax Standard Imaging electrometer. Field Emission Scanning Electron Microscopy (FESEM, MIRA3 TESCAN) equipped with energy-dispersive X-ray EDX, and X-ray diffraction patterns (XRD, Philips diffractometer, PW1800) were carried out in Razi metallurgical research center in Iran. Atomic force microscopy (AFM, Easy Scan2, Nanosurf) was accomplished in Beam Gostar Taban Laboratory-Tehran, Iran), Transmission Electron Microscopy (TEM, Philips CM30, 200 kV Netherlands) of the fractured surfaces of the samples were performed at Tehran University of Iran. Fourier Transform Infrared Spectroscopy (FTIR, TENSOR 27-BRUKER) was conducted at the wavenumber rage of 400–4000 cm^−1^ via the transmittance mode available at Nuclear Science and Technology Research Institute-Iran. X-ray photoelectron spectroscopy (XPS, Specs-FlexPS) and differential thermal gravimetric analysis (TGA/DTG, TA Instrument SDT Q600) were done in the central laboratory of Isfahan-Iran to study the surface properties and thermal stability of the samples, respectively.

## Results and discussion

### Characterization

#### FESEM-EDX mapping

Figure 1B shows the FESEM image of the fractured surface of B_60_ sample, while the elemental maps of Carbon (C), Oxygen (O), and Bismuth (Bi) along with the corresponding overlay are depicted in Fig. 1c–f. As can be seen from Fig. 1b, the Bi_2_O_3_ nanoparticles are dispersed in the HDPE matrix in the separate isolated regions attributed to semi-crystalline nature of the polyethylene. Although it has been proven that higher degrees of polymer crystallinity hinder nanoparticle dispersion at higher concentration of the inclusions which leads to agglomeration of the nanoparticles^26^. Impressively, the nanocomposite contains areas that are evenly distributed in a cauliflower-like structure. This structure has been observed in other studies^27,28^.

The quantitative EDX analysis of the surface elements of the B_60_ sample are shown in Fig. 1g, and Table 2. The absorption edges of bismuth (M_α_ and M_β_) and other elements, such as carbon and oxygen (K_α_), emerged as fingerprints within the selected energy range, confirming the presence of bismuth in the synthesized sample. The fact that the weight percentage of bismuth in Table 2 for the B_60_ sample containing 60 wt% Bi_2_O_3_ is estimated at around 10.5 w% proves the probability of agglomeration formation in this sample. This is related to the fact that polyethylene is a semi-crystalline polymer and nanoparticles tend to disperse more in the amorphous regions than in the crystalline regions^1,26^.

**Table 2:** FESEM-EDX values of Carbon, Oxygen, and Bismuth in the area where EDX mapping was performed in Fig. 1b.

Element	Line	Intensity	wt %	Area%
C	Kα	487.7	86.14	96.49
O	Kα	7.3	3.37	2.84
Bi	Mα	89.4	10.49	0.68
			100.00	100.00

#### AFM

**Figure 2:**
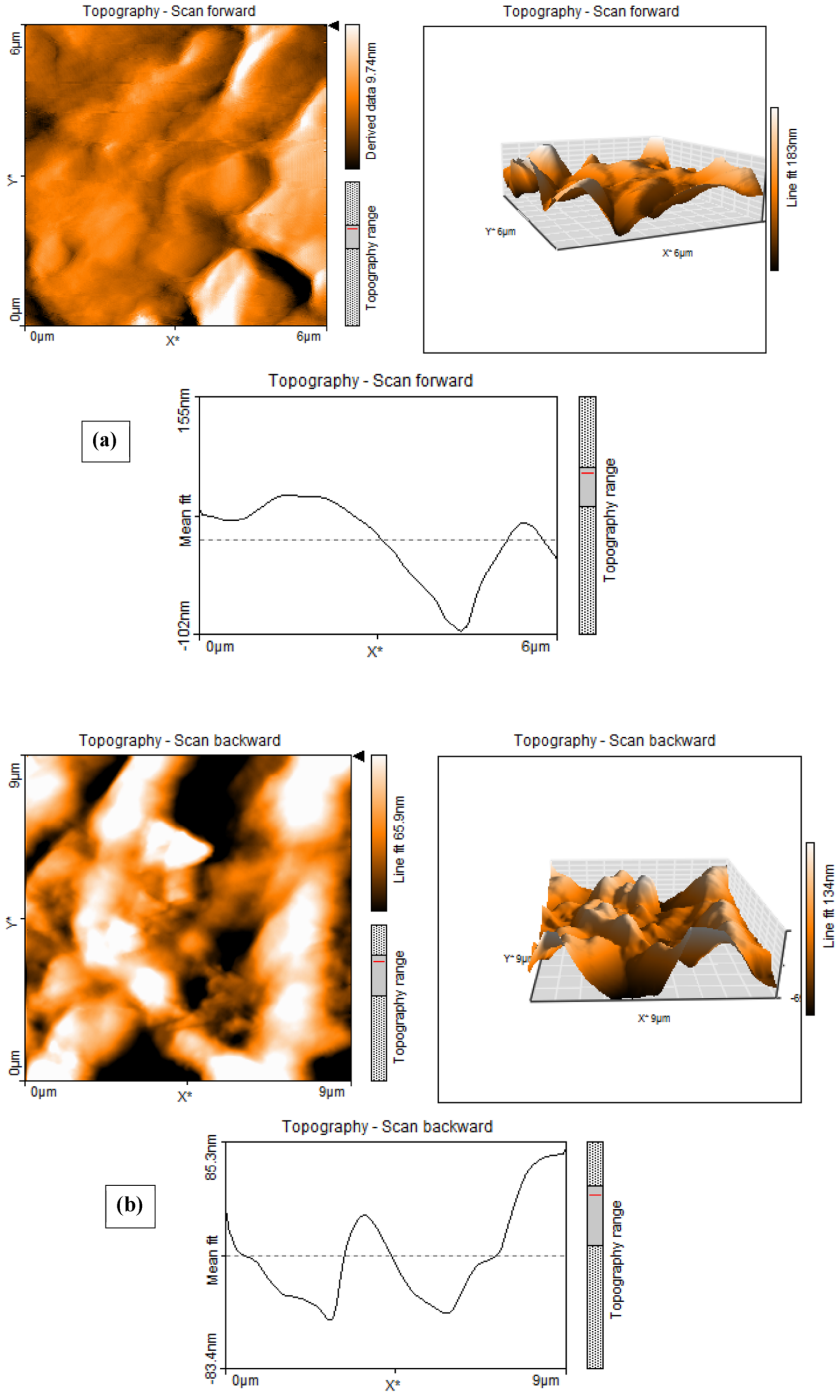
AFM analysis of (**a**) B_0_, and (**b**) B_60_ samples.

AFM is a microscopic technique for imaging a surface topography by using attractive and repulsive interaction forces between a few atoms attached at a tip of a cantilever and a sample which plays an important role to manipulate and position the nanoparticles^29–31^. AFM analysis of B_0_ and B_60_ samples is illustrated in Fig. 2 exhibiting the topographic images at ambient conditions in non-contact mode. Although, it is difficult to discuss about the size of the Bi_2_O_3_ particles from the AFM image due to agglomeration created in the 60 wt% in Fig. 2b, considering the peak height (z direction), it can be mentioned that the size of these particles, at least in one dimension are consistent with their specifications in the catalogue, namely between 90 and 210 nm.

#### TEM

**Figure 3:**
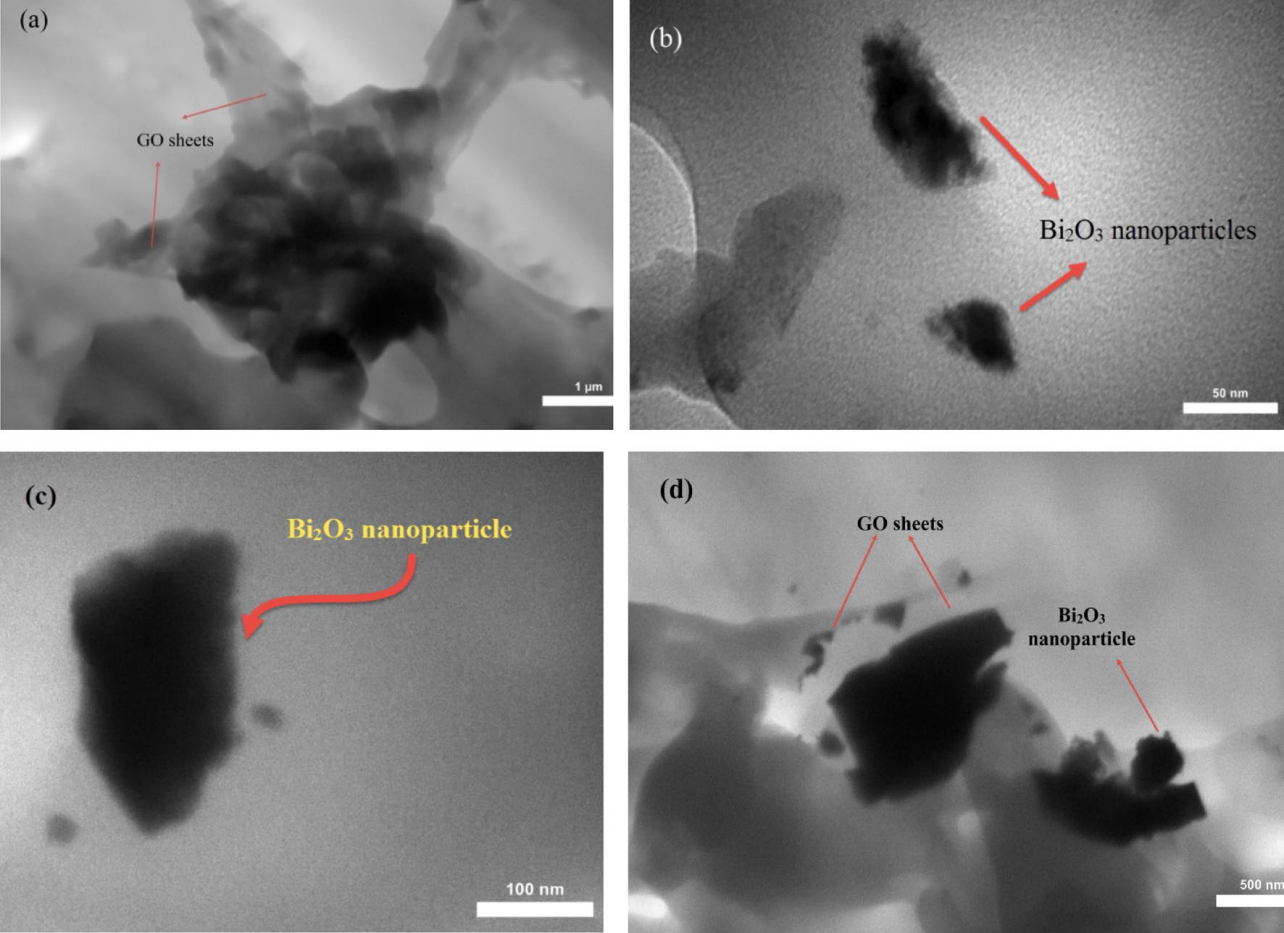
TEM images of the B_60_ sample at two magnifications exhibiting the Bi_2_O_3_ nanoparticles and GO sheets.

Figure 3 depicts the TEM images of the fractured surface of the B_60_ sample. To perform TEM tests, Ultramicrotomy was carried out by a Reichert-Jung Ultracut-E supplied by a Diatome 45º diamond knife. TEM image confirms the dimensions of the Bi_2_O_3_ nanoparticles in agreement with the prepared nanoparticles in accordance with their catalogue, namely 90–210 nm. Also, the TEM image of graphene oxide nanosheets illustrates the flake-like shapes of graphene oxide, which is in agreement with Song et al.’s findings^32^. According to Fig. 3, presence of the Bi_2_O_3_ nanoparticles in the HDPE matrix is evident, despite the low weight fraction of the GO sheets with micrometer size in the ternary nanocomposite. According to Fig. 3d, the wrinkled edges of the GO nanosheets are clearly visible.

#### XRD analysis

**Figure 4:**
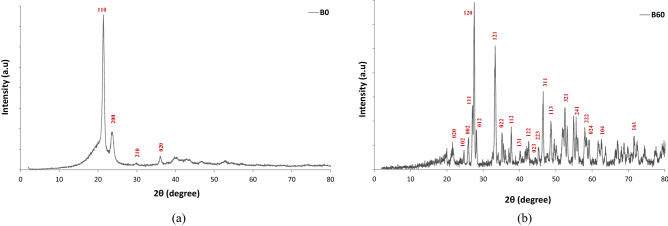
XRD analysis for B_0_, and B_60_ samples.

As shown from Fig. 4, XRD analysis of the B_0_ and B_60_ samples is exhibited, indicating the presence of the semi-crystalline structure of the samples. According to Fig. 4a, for pure HDPE (B_0_ sample), there are several reflection peaks at 2θ of 21.5, 23.9, 29.3 and 35.9° that correspond to the (110), (200), (210) and (020) planes, respectively in accordance with the standard JCPDS file number 00-040-1995^33,34^. As shown in Fig. 4b, XRD pattern for the B_60_ sample demonstrates various characteristics peaks for Bi_2_O_3_ at 2θ of 21.6° (020), 24.6° (102), 25.9° (002), 27.0° (111), 27.5° (120), 27.7° (012), 33.2° (121), 35.1° (022), 37.7° (112), 40.0° (131), 42.5° (122), 44.1° (023), 45.1° (223), 46.5° (311), 48.6° (113), 52.4° (321), 54.8° (241), 57.9° (222), 58.5° (024), 62.5° (104) and 71.4° (161). The peaks were in good agreement with the standard JCPDS file number 76-1730^3,35,36^.

#### XPS analysis

**Figure 5:**
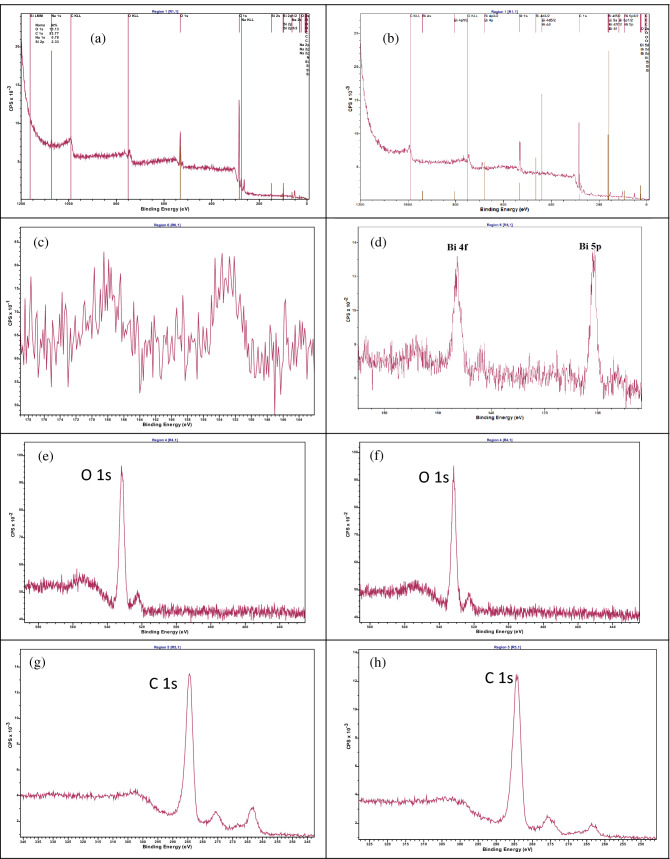
XPS analysis for B0, and B_60_ samples. Survey spectra for (**a**) B_0_, and (**b**) B_60_, (**c**) peaks between 140 and 180 eV for B_0_, and (**d**) Bi main peaks for B_60_, O 1 s core-level for (**e**) B_0_, (**f**) B_60_, C 1 s core-level for (**g**) B_0_, and (**h**) B_60_ sample.

XPS analysis is widely used to understand surface properties when analyzing carbon materials^37^. As depicted in Fig. 5, the chemical binding energy of B_0_, and B_60_ samples was quantified by XPS analysis at ambient temperature. The main peak at 529.2–529.7 eV could be attributed to lattice oxygen^38^. As can be seen from Fig. 5d, the peaks corresponding to the Bi can be distinguished at the binding energies between 158.6 and 159.1 eV in accordance with the literature data^38–40^. All carbonaceous materials, including GO, have a C 1 s peak for sp^2^ carbon with a binding energy of 284.5 eV, whereas the sp^3^ sample was acquired to have a C 1 s energy of 285.0 eV^37^. Figure 5g,h exhibit C 1 s core-level for B_0_ and B_60_ samples respectively. Via the obtaining of C 1 s, and O 1 s, core-levels and Bi spectra in the XPS analysis of the under study samples in this research, results confirmed the presence of bismuth particles in the nanocomposite regarding the binding energy for backscattered electrons from the samples.

#### FTIR analysis

**Figure 6:**
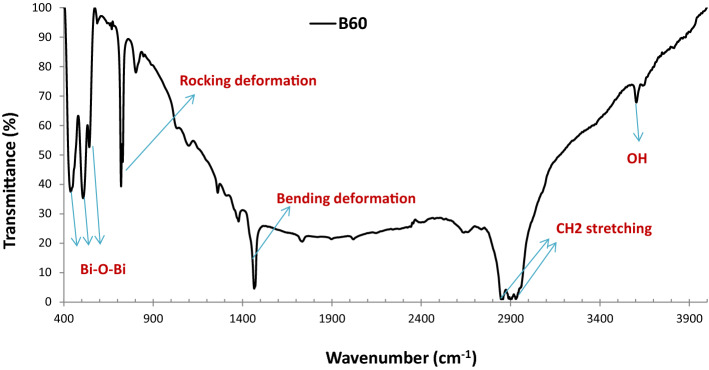
FTIR spectrum of the B_60_ sample exhibiting various functional groups and elements.

In order to investigate the chemical composition of the B_60_ sample, the FTIR analysis was performed at the wavenumber range of 400–4000 cm^−1^ via the transmittance mode. According to Fig. 6, the most important polyethylene bands at 650–750 cm^−1^ are related to rocking deformations, bending deformation at 1400–1550 cm^−1^ and CH_2_ stretching at 2800–3000 cm^−1^, which are in accordance with findings of Gulmine et al.^41^. The main bands at 400–700 cm^−1^ are pertinent to the bismuth-oxygen or Bi-O-Bi vibration and the stretching vibration of the hydroxyl group or OH in the range of 3200–3600 cm^−1^, which is in agreement with the previous works^42–44^.

#### TGA/DTG analysis

**Figure 7:**
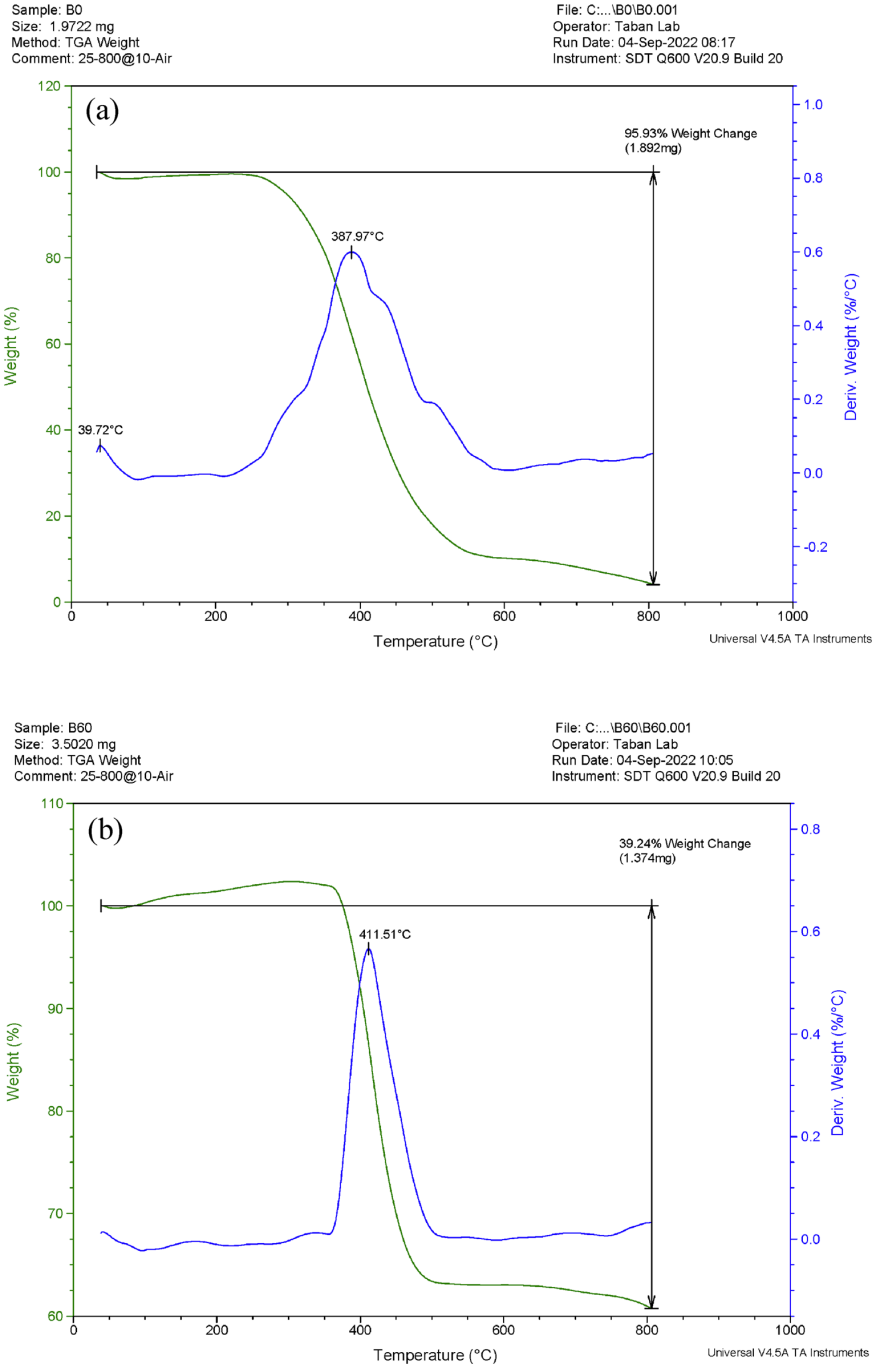
Examination of the thermal stability for (**a**) B_0_ and (**b**) B_60_ samples by the TGA/DTG analysis.

As depicted in Fig. 7, TGA/DTG analysis was conducted at air atmosphere and heating rate of 10 °C/min to examine the thermal performance of the two B_0_ and B_60_ samples. Thermal features are implemented in the range of 35 to 800 °C. The decomposition temperature obtained from the TGA analysis provides a measure of thermal stability. As can be seen in Fig. 7, the initial decomposition temperature of the B_60_ (at 373 °C) was found higher than B_0_ (at 256 °C). The maximum weight changes between the temperatures of 35–800 °C were measured at 95.9% and 39.2% for B_0_, and B_60_, respectively. It can be mentioned that for B_60_ sample compared to B_0_, the rate of weight loss decreases. Also, based on the DTG analysis, glass transition temperatures (T_g_) of B_0_, and B_60_ were measured at 387.97 °C, and 411.51 °C respectively. It seems that addition of Bi_2_O_3_ nanoparticles into the HDPE matrix at concentration of the 60 wt% has improved the flame retardancy of the composite material. So, adding the Bi_2_O_3_ nanoparticles in the HDPE matrix, leads to improving the thermal stability of the nanocomposite at higher temperature ranges in comparison with the pure HDPE.

### Quantum efficiency

At certain energy, there is a direct relationship between the mass attenuation coefficient of a material and the sensitivity to radiation as a detector or dosimeter. Thus, quantum efficiency (QE) is introduced via the Eq. (1) ^8^:

1 $$QE = \left( {1 - e^{{ - (\frac{\mu }{\rho })^{{}} \rho x}} } \right)^{{}} 100\%$$

In which µ/ρ is the mass attenuation coefficient, obtained from a photon cross sections database^45^, while ρ and x are the density and thickness of the composite material, respectively.

**Figure 8:**
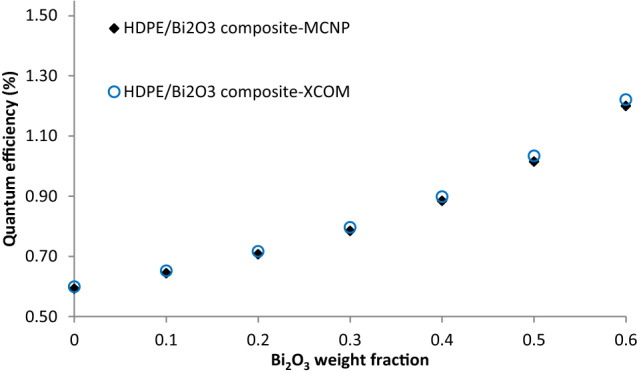
Calculated quantum efficiency of HDPE/Bi_2_O_3_ composites with thickness of 1 mm in various concentrations.

Figure 8 shows the calculated quantum efficiency of HDPE/Bi_2_O_3_ composite with 1 mm thickness at various concentrations through the MCNP code and XCOM programs at 1250 keV^45,46^. As can be seen from Fig. 8, the QE increases exponentially, rising by a factor of 2 as the concentration of the Bi_2_O_3_ particles increases from 0 to 60 wt%. This figure demonstrates that the maximum efficiency for these composites at 1250 keV is obtained as 0.6% to 1.2% for 60 wt% of Bi_2_O_3_ inclusions. Although the efficiency values for high-energy photons are relatively low, these composites as promising materials can be used to measure the low-energy x-rays and photons at the diagnostic level, because the probability of photoelectric absorption event is high at low energies for high-Z absorber materials as Z^n^/E^3.5^, in which Z is atomic number of the absorber material, E is the photon or x-rays energy, and n is a variable exponent between 4 and 5^47^. In general, the quantum efficiency of the composites can be increased by adding high atomic number heavy metal oxide inclusions into the polymer matrix.

### Bias-polarity, linearity and signal-to-noise ratio

At the beginning, it is reminded that the response of the dosimeter studied in this research is the change of electric current during irradiation. Firstly, in order to explore the bias polarity dependence of the dosimeter response, the positive bias was selected at + 100 V using the electrometer. Then, to measure the dark current (current in the absence of radiation) at different voltages, electric charge was collected and measured in a time interval of 15 s. Afterward, the dosimeter was exposed to gamma-rays of ^60^Co using the Picker-V9 machine at the SSD = 80 cm for one minute, and the amount of electric charge was measured in 15 s intervals. By dividing the collected charge by the measurement time, the amount of dark current (I_0_) and total current (I_tot_) was measured and the average of the quantities was calculated for repeated four measurements during one minute. Finally, the amount of photocurrent (I = I_tot_−I_0_) was measured at the desired voltage. This process was repeated in positive bias from + 100 V to + 1000 V voltage at the SSD = 80 cm. In the next phase, the negative bias was selected and voltages from − 100 V to − 1000 V were applied to the dosimeter and the photocurrent was recorded at each voltage.

**Figure 9:**
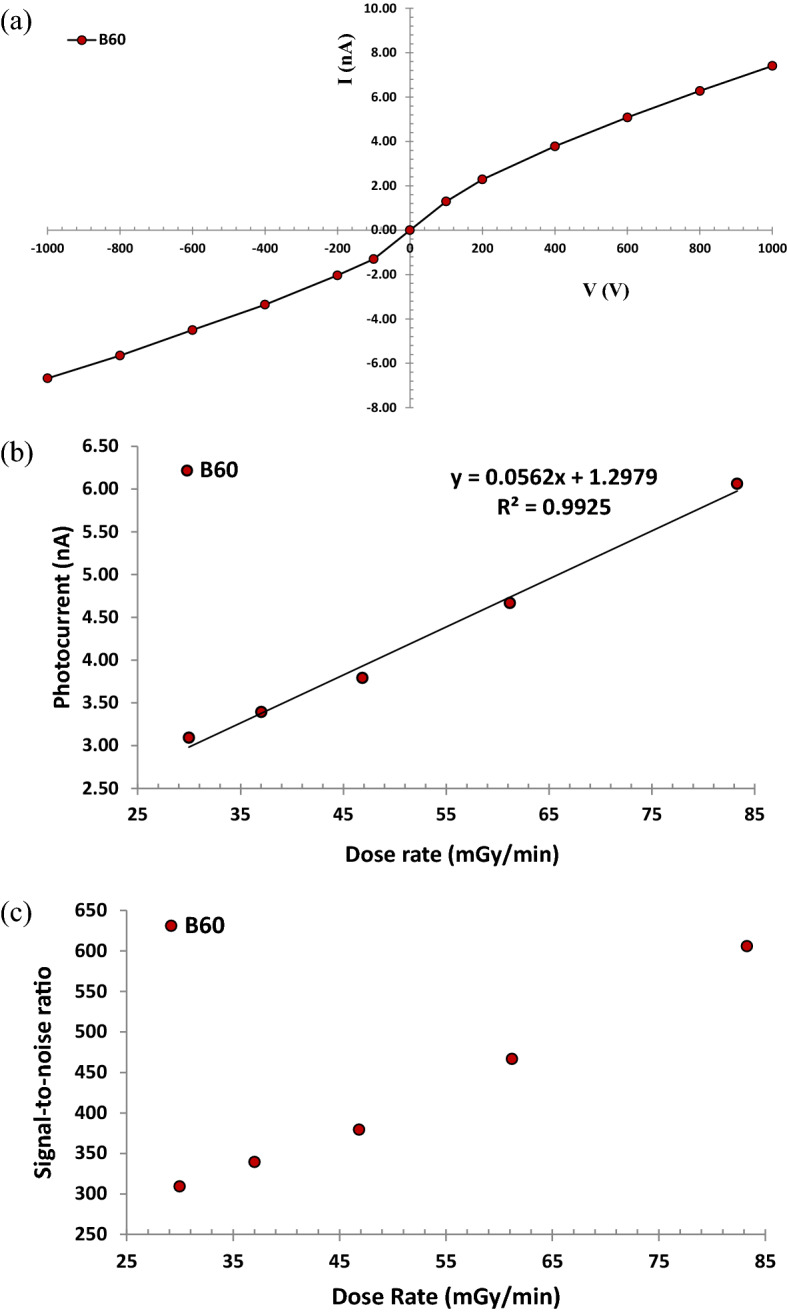
Investigation of (**a**) current-voltage at a fixed-dose rate of 46.85 mGy/min using Picker-V9 exhibiting maximum 1.5% standard deviation (1σ), (**b**) linearity response of the dosimeter 1.5% (1σ), and (**c**) signal-to-noise ratio for B_60_ sample at 400 V, 1.1% (1σ).

In Fig. 9a, the current-voltage (I-V) curve is exhibited for the B_60_ sample at 46 mGy/min and SSD = 80 cm. The I-V measurements of the nanocomposite in a constant dose rate exhibit that the response is approximately linear within the ± 1000 V. Generally, the optimum bias voltages for the semiconductor detectors are near the saturation region. The radiation response of the nanocomposites until ± 1000 V was not saturated. This is not a challenging issue, because if radiation of single energy and type are involved, it is sometimes possible to operate the detector at a bias voltage that is short of true saturation without significant deterioration in the energy resolution. After all, the fraction of charge lost for each event is likely to be nearly constant^47^. For the Bi_2_O_3_-HDPE nanocomposite as a solid state dosimeter, the atomic density is much higher than free-air ionization chambers or Geiger–Müller counters. So, it is expected that we have more ionizations in this dosimeter. Also, there is possibility occurrence of Bremsstrahlung radiation in this nanocomposite material, which increases the response of the dosimeter and working voltage in comparison with dosimeters containing gas or air in their sensitive volumes. Also, the saturation of the dosimeter response, depends on the absorbed dose or dose rate. As shown in Fig. 9a, the dose rate is 46.85 mGy/min, which shows that the response of the dosimeter is not saturated until the voltage of ± 1000 V. Maybe the saturation event can be observed at higher doses or dose rates. Due to limitations in the activity of the gamma-ray sources applied in this study, the saturation phenomenon did not occur until the dose of 166.04 mGy/min. Basically, in ionization chambers, there is a polarity effect, and the response needs a polarity correction factor. Error bars for the I-V curve showed less than 1.5% discrepancy in the standard deviation of the measured photocurrent. As shown in Fig. 9a, at the voltages of ± 1000 V, in bias-polarity point of view, the maximum discrepancies for the B_60_ sample were measured as 9.9%, respectively, which proves that at higher voltages, the dosimeter response may depend on its polarity. At 400 V, a common voltage for Geiger counters, the maximum discrepancies for the B_60_ sample were measured as 11.1%, respectively.

One of the characteristics of a good real-time dosimeter is the linearity of its response in a wide range of dose rates. In order to examine the linearity of the dosimeter response in this research, the nanocomposite dosimeter was subjected to a constant voltage of 400 V at different source-surface distances (SSDs) in the SSDL of Iran-Karaj, and the amount of photocurrent was measured four times in each SSDs. It is worth noting that the amount of dose rate in the standard radiation fields of the SSDL laboratory at different distances is quite specified. Figure 9b shows the average photocurrent vs. dose rate for the B_60_ sample. The value of R^2^ = 0.9925 with linear regression indicates the linearity of the dosimeter response ranging from 30 to 83 mGy/min exhibiting a maximum 1.5% standard deviation (1σ) in the measurement. As can be seen in Fig. 9b, more ionization and consequently more holes are expected to be created in the sensitive volume of the dosimeter as the dose rate increases, and these factors contribute to increase in the dosimeter response. In fact, in a polymer-heavy metal oxide nanocomposite, some parameters can assess the amount of sensitivity, including thickness, heavy metal oxide wt%, applied bias voltage, the crystallinity of the polymer matrix, and so on. It should be mentioned that typical doses are between 12 and 97 Gy for diagnostic and therapeutic radiation applications^9,48–50^. For external radiotherapy, the total absorbed dose is fractionated in several sessions. Thus, the dose rate can rely on the order of mGy per minute for each session. It should be noted that the dose response of a ternary nanocomposite is directly related to the sensitive volume of the dosimeter, so that by decreasing the sensitive volume of the dosimeter, the dose response is significantly reduced.

Another characteristic of a good dosimeter is its high sensitivity in the radiation fields. To measure the sensitivity of the nanocomposite dosimeter in this research, the value of this quantity can be determined according to the slope of the photocurrent-dose rate plot. The slope of this line has a dimension of nA/(mGy/min). By converting the unit of minute to second, the unit is converted to 60 nC/mGy. At the end, by multiplying this quantity by the slope value of the I-Dose rate plot, the sensitivity value of the dosimeter can be determined. This method has also been used in some references and previous works^1,8^. Thus, sensitivity of the B_60_ sample to gamma-rays of ^60^Co using Picker-V9 irradiator was obtained at a fixed voltage of 400 V considering the slope of the I-Dose rate plots in Fig. 9b, which is evaluated in Table 3, accordingly. It can be found that the sensitivity of the B_60_ sample is more than eight times greater than the PIN diode dosimeter at the therapy level which was studied by Kumar et al.^51^. Also, the sensitivity of the B_60_ nanocomposite dosimeter in this research is significantly higher than the conventional dosimeters in radiotherapy, whose sensitive volume is often filled with free-air.

**Table 3:** Linearity response of the B_60_ sample and comparison its sensitivity with a PIN diode dosimeter.

Sample	Trendline	R^2^	Sensitvity (nC·mGy^-1^)	Ref
B_60_	y = 0.0562x + 1.2979	0.9925	3.4	This work
PIN diode	–	–	0.4	^51^
Farmer chamber (Type 30,010)	–	–	0.02	^52^
Roos (TN34001)	–	–	0.012	^52^
Advanced Markus (TN34045)	–	–	0.0067	^52^
Semiflex (TN31013)	–	–	0.01	^52^

Another characteristic of a good dosimeter is a high signal-to-noise ratio (the amount of net current to the dark current) in a constant dose rate. So, to measure the quantity of signal-to-nose-ratio related to the nanocomposite dosimeter under gamma irradiation at different SSDs, the average photocurrent during the irradiation for one minute was divided by the value of average dark current. As shown in Fig. 9c, the signal-to-noise ratio of the B_60_ sample at 400 V against the gamma-rays of Picker-V9 is depicted. As illustrated from Fig. 9c, with increasing the dose rate ranging from 30 to 83 mGy/min, the signal-to-noise ratio will be increased by 309 to 606 times compared to the dark current. This phenomenon may be pertinent to the emission of secondary electrons, excitation, ionization, and Bremsstrahlung radiation during the interaction of the gamma-rays with the atomic structures of the nanocomposites.

It should be noted that the amount of electrical conductivity of the sensitive volume is a crucial factor for a dosimeter. Thus, the amount of dark current should be on the order of picoamps (pA). Therefore, to prevent the electroforming phenomenon (migration of metal particles to the sensitive volume)^17^, the electrical resistance of the sample was measured at room temperature with a two-probe a Digital Insulator Tester MIS-3D. For B_60_ sample, the average initial amount of dark current (current in the absence of irradiation) at the fixed voltage of 400 V was measured as 5 pA.

### Time evolution, stability and repeatability

**Figure 10:**
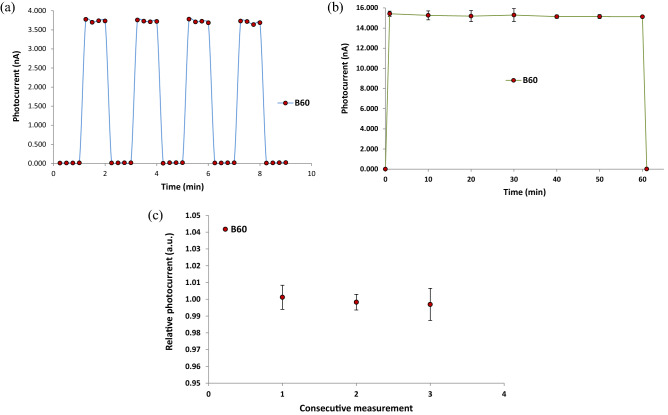
Examination of (**a**) time evolution of the dosimeter response at a fixed-dose rate of 46.85 mGy/h, (**b**) Stability at 166.04 mGy/min, during 1 h irradiation, and (**c**) Repeatability for B_60_ sample at a same dose rate of 46.85 mGy/min and 400 V exhibiting maximum 1% standard deviation (1σ).

In order to check the response of the nanocomposite dosimeter at a fixed distance and in different time intervals, the time evolution diagram was plotted. For this purpose, at SSD = 80 cm with dose rate of 46.85 mGy/min using the Picker-V9 machine, first, the amount of dark current was measured for 1 min in the 15 s intervals. Then, the nanocomposite dosimeter was exposed to radiation for 1 min and amount of the photocurrent was recorded. Again, the irradiation was stopped and dark current was measured for 1 min. Then the irradiation was repeated for 1 min. This process of turning on and off the irradiation system and recording the amount of dark current and photocurrent was repeated in four cycles. In Fig. 10a, the time evolution of the dosimeter response for B_60_, is exhibited using the Picker-V9 machine at the constant dose rate 46.85 mGy/min. The average dark current and photocurrent at the fixed voltage of 400 V were measured as 5 pA, and 3.721 nA respectively.

Another characteristics of a good dosimeter is the long-term stability of the dosimeter response. Therefore, to investigate the long-term stability of the dosimeter response, as shown in Fig. 10b, the B_60_ sample was exposed to gamma-rays of the ^60^Co Theratron-780 machine at a fixed SSD = 80 cm with dose rate 166.04 mGy/min, field size 30 × 30 cm^2^, and 400 V for 1 h, in which the photocurrent values were measured in ten-minute intervals using the electrometer. Results show that the dosimeter response during the 1 h irradiation is stable as 15.226 ± 0.100 nA with 0.66% standard deviation. It should be noted that GO degradation/reduction may occur at higher doses at the order of kGy^53^. In future studies, it is recommended to test the stability of this dosimeter in therapeutic-level high-energy photon accelerators. The precision and the repeatability of the dosimetry results are very important for a typical dosimeter. Therefore, to examine the repeatability of the measurement results using the nanocomposite dosimeter, the amount of photocurrent at a fixed distance of SSD = 80 cm from the Picker-V9 machine was measured three times in the consecutive 15 s intervals using the electrometer at a fixed voltage of 400 V. Figure 10c shows the plot of three consecutive relative readings related to B_60_ sample. The plot indicates that the response of the dosimeter is reproducible within 0.97%, which is well within the acceptable range of radiation therapy applications. Thus, in this work, the repeatability of the dosimeter response is less than 1%.

### Angular, field size and energy dependence

One of the features of an ideal dosimeter is the independence of its response from the radiation angle with the surface of the dosimeter. In order to investigate the angular dependence of the nanocomposite dosimeter response, the Picker-V9 irradiation system was used in the horizontal mode. For this purpose, the nanocomposite dosimeter was attached to the surface of the standard PMMA slab phantom of size 30 × 30 × 15 cm^3^, and the phantom was placed on a rotating scale plate made of polyethylene with a motor capable of rotating in both directions in front of the beam at a distance of 98 cm with a dose rate of 30.672 mGy/min. At each angle, the amount of photocurrent was measured four times in the 15 s intervals using the electrometer under 400 V. The measurements were repeated for different rotation angles from 0 to 45° with a step of 15° in both positive and negative directions at a fixed distance.

**Figure 11:**
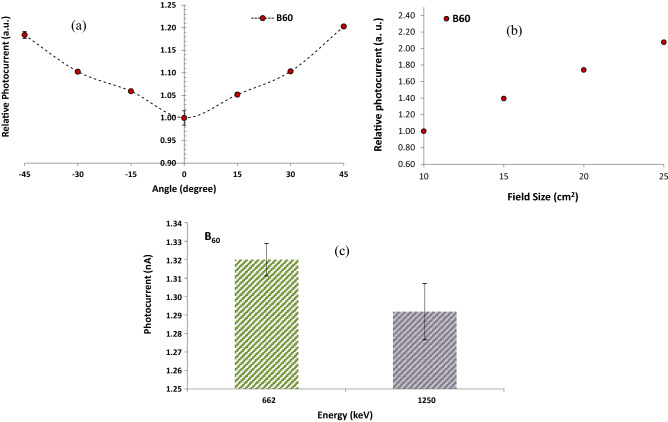
Evaluation of (**a**) angular dependence in various gamma-ray incident angles exhibiting a maximum 1.6% standard deviation (1σ), (**b**) field size dependence exhibiting a maximum 1% standard deviation (1σ), and (**c**) energy dependence at a constant dose rate of 1.83 mGy/min related to the ^137^Cs and ^60^Co sources for B_60_ sample.

Figure 11a shows the angular dependence of the B_60_ sample irradiated at SSD = 98 cm, dose rate = 30.672 mGy/min, field size = 10 × 10 cm^2^ using the Picker-V9 machine. The average photocurrents in various angles, namely θ =  ± 45° were measured and normalized to θ = 0°. Results showed that at θ = 45°, the dosimeter response was about 20% higher than the zero angle. However, at higher SSDs, angular dependency may be reduced. As can be seen from Fig. 11a, the angle of the incident radiation plays an essential role in the process of measurement. As the beam angle increases on the surface of the nanocomposite sample, more ions are created diagonally in the sensitive volume of the material^54^. So, the amount of photocurrent increases which means that the efficiency of radiation measurement is dependent on the angle of the incident radiation.

For a dosimeter used in the radiotherapy, the response of the dosimeter depends on the size of the radiation field. In routine dosimeters such as ionization chambers, the response of the dosimeter is often normalized to one in the radiation field of 10 × 10 cm^2^ at the standard distance of SSD = 80 cm. To measure the response of the dosimeter in other field sizes, the normalized dose rate is multiplied by a correction factor considering the field size. In this research, to investigate the response of the nanocomposite dosimeter in the radiation field with different sizes, the ^60^Co source of the Picker-V9 machine was used in the vertical mode. At first, the response of the dosimeter, i.e., the change in electric current (photocurrent) was measured in field size of 10 × 10 cm^2^ at the SSD = 80 cm under a fixed voltage of 400 V; Then this process was repeated for other radiation field sizes, i.e. 15 × 15 cm^2^, 20 × 20 cm^2^ and 25 × 25 cm^2^ at the fixed SSD = 80 cm, and 400 V.

Figure 11b shows the radiation field size dependence of B_60_ sample at a fixed SSD = 80 cm using the Picker-V9 ranging from 10 × 10 cm^2^ up to 25 × 25 cm^2^. The figure shows the response change of the dosimeter with the field size for the ^60^Co gamma-ray beam, which exhibits a good agreement with Kumar’s findings of the PIN diode dosimeters at the therapy level^51^. Figure 11b demonstrates that the dosimeter response improves significantly as the field size increases. To justify this event, it can be mentioned that since in this research, the sensitive volume of the dosimeters is significantly larger than PIN diode dosimeters^51^, it is probable as the radiation field size increases, the scattered radiations around the dosimeter will be read by the dosimeter subsequently. Therefore, to solve this issue, it is recommended to use a water phantom or tissue-equivalent plastic phantom at the reference point instead of measuring in air.

One of the aspects of a reliable dosimeter is independence of the dosimeter response to the incident beam energy. To investigate the energy dependence of the dosimeter response for B_60_, two sources of ^60^Co (Picker-V9) and ^137^Cs (OB-85) with energies of 1250 keV and 662 keV at the same dose rate of 1.83 mGy/min were used at the SSDs of 389 cm and 60 cm, respectively. For each energy, the response of the dosimeter, i.e. photocurrent, was recorded in 1 min at the 15 s intervals. It is worth mentioning that the nanocomposite dosimeter was attached on the surface of a standard PMMA phantom and the phantom was placed in front of the beam at the certain distances.

As can be seen from Fig. 11c and Table 4, the dosimeter response exhibits 2.2% difference for various energies. Since the high-energy photon beams operates with maximum electron energy between 4 to 25 MV^55^, to investigate the energy dependence of this class of dosimeters in future researches, it is suggested to use high-energy photon accelerators at the radiotherapy level in different energies.

**Table 4:** Investigation of the energy dependence of the dosimeter response at the same dose rate of 1.83 mGy/min for B_60_ sample.

Radiation source	Energy (keV)	Photocurrent (nA)
^137^Cs	662	1.32 ± 0.01
^60^Co	1250	1.29 ± 0.02

### Effect of reinforcement phase loadings

After evaluation of the dosimetry characteristics of the Bi_2_O_3_/HDPE nanocomposite containing of 60 wt% Bi_2_O_3_ and 0.1 wt% GO, effect of additives namely Bi_2_O_3_ and GO nano-fillers on the dosimetry response were investigated at various concentrations of the inclusions (Supplementary Notes 2 and 3, Figs. 2E–4E). Firstly, effect of Bi_2_O_3_ wt% on the dosimetry response of the samples was investigated in different concentrations of 0, 20, 40, and 60 wt% and fixed amount of 0.1 wt% for GO at various dose rates. Results showed that as the Bi_2_O_3_ wt% increased, the dosimeter response enhanced subsequently. It was also concluded that at high-weight fractions of the Bi_2_O_3_ nano-fillers near 60 wt%, because of the semi-crystalline nature of the polyethylene matrix, agglomeration occurs (Fig. 1b) and the dosimeter response decreases.Secondly, effect of adding GO on the dosimetry response of the samples was investigated in different concentrations of 0, 0.1, 0.5, 1 and 2 wt% and fixed amount of 40 wt% for Bi_2_O_3_ at various dose rates. Results revealed that as the GO wt% increased, the dosimeter response enhanced remarkably. Results indicated that the correct selection of the inclusions namely heavy metal oxide and graphene oxide nano-fillers played an important role in the dosimetry response of such nanocomposites.

Here in, some facts about the physics of radiation detection are exhibited. Events such as the photoelectric effect, Compton scattering, and pair production (for photons with energies above 1.022 MeV) can occur stochastically as a result of the interaction of high-energy photons with matter. Although the dosimeter material studied in this project show the occurrence of the all events, Compton scattering appears to be the dominant phenomenon, as the probability of the photoelectric effect decreases with increasing the photon energy. That is, the photons lead to generation of the secondary electrons within the dosimeter material. The secondary electrons are converted to the signal by applying a suitable voltage on the dosimeter. It should be noted that secondary electrons can generate bremsstrahlung radiation near the heavy nuclei of the bismuth nanoparticles which leads to enhance the sensitivity of the dosimeter.

## Conclusion

In this experimental work, dosimetry characteristics of a ternary nanocomposite based on the HDPE/Bi_2_O_3_/GO material containing 60 wt% Bi_2_O_3_ and 0.1 wt% GO was investigated in a ^60^Co radiation field at the therapy level over the dose rate of 30–254 mGy/min. A solution method was used for fabricating the sample. XRD, XPS, AFM, FESEM-EDX mapping, TEM, FTIR, and TGA/DTG analyses were performed on the samples. XRD analysis showed a semi-crystalline behavior for the nanocomposite. AFM, FESEM, and TEM analyses confirmed the presence of the inclusions in the nanocomposite exhibiting a cauliflower-like structure of the material. The XPS analysis through the C 1 s, and O 1 s, core-levels and Bi spectra in samples confirmed the presence of bismuth particles in the nanocomposite. FTIR results showed that the main bands at 400–700 cm^−1^ were pertinent to the bismuth-oxygen or Bi-O-Bi vibration, the most important polyethylene bands at 650–750 cm^−1^ were related to rocking deformations, bending deformation at 1400–1550 cm^−1^ and CH_2_ stretching at 2800–3000 cm^−1^, which were in accordance with other works in literature. TGA/DTG analysis showed that for B_60_ sample compared to B_0_, the rate of weight loss decreased significantly. Results exhibited that adding 60 wt% Bi_2_O_3_ to the HDPE matrix led to increase the glass transition temperature from 387.97 °C to 411.51 °C, and enhancing the thermal stability of the nanocomposite. After fabrication and characterization of the samples, several dosimetric characteristics were carried out on the B_60_ sample, namely linearity, angular dependence, energy dependence, bias-polarity, field size, and repeatability of the data. Results exhibited a linear behavior at the range of 30–254 mGy/min. The sensitivity of the B_60_ sample was measured as 3.4 nC mGy^−1^, which was eight times higher than ordinary PIN diode detectors. The angular response variation was about 20% for normal beam incidence. However, at higher SSDs, angular dependency may be reduced. The dosimetry response was energy independent with 2.2% discrepancy. Bias-polarity experiment showed that the dosimeter response was dependent on bias voltage with 11.1% maximum discrepancy. The field size test showed that due to the high sensitivity of the sample to backscattered radiations, the dosimetry response is significantly dependent on the radiation field size, which confirms the need for measurement and dosimetry inside the phantom at the reference depth. The repeatability of the dosimeter response was less than 1%. Also, the dosimeter response during the 1 h irradiation was stable with 0.66% standard deviation. It was also concluded that at high weight fractions, around 60 wt%, aggregation occurred (which confirmed in the FESEM image in Fig. 1b, and also AFM analysis) and the dosimeter response decreased due to the semi-crystalline nature of the polyethylene matrix.

Effects of additives namely Bi_2_O_3_ and GO nano-fillers on the dosimetry response were investigated at various concentrations of the inclusions (Supplementary Notes 2 and 3), in which results evidenced that the correct selection of the inclusions namely bismuth oxide and graphene oxide nano-fillers played an essential role in the dosimetry response, which could be used for the real-time dosimetry of the photon fields at the therapy level.

## Supplementary Information

Supplementary Information.

## Data Availability

The datasets used and/or analysed during the current study available from the corresponding author on reasonable request.
