# Supplementary material for: Evaluation of dosimetric characteristics of a ternary nanocomposite based on High Density Polyethylene/Bismuth Oxide/Graphene Oxide for gamma-rays
Source: Sci Rep. 2022 Nov 5;12:18798. doi: 10.1038/s41598-022-23605-y (PMC9637186; doi:10.1038/s41598-022-23605-y)
Supplement: Supplementary file 1 — Supplementary Information. [file 41598_2022_23605_MOESM1_ESM.docx]

**Evaluation of dosimetric characteristics of a Ternary nanocomposite based on High Density Polyethylene/Bismuth Oxide/Graphene Oxide for gamma-rays**

Amir Veiskarami^1^, Dariush Sardari^1^, Shahryar Malekie^2^*, Farshid Babapour Mofrad^1^, Sedigheh Kashian^2^

^1^ Department of Medical Radiation Engineering, Science and Research Branch, Islamic Azad University, Tehran, Iran

^2^ Radiation Application Research School, Nuclear Science and Technology Research Institute, P.O. Box 31485-498, Karaj, Iran

*smaleki@aeoi.org.ir

**Supplementary Note 1**

Junctions of the electrodes are of extreme importance. In this research, copper electrodes with thickness of with 100 μm were selected, in which were coated on the samples using silver paste with high electrical conductivity and excellent adhesive properties. For electrode production, the copper electrode surface in front of the incident gamma rays was examined at 3.5×3.5 cm^2^. While, the surface of the sample was 4×4 cm^2^; therefore, the top copper electrode did not coat the whole surface, and a part of the sample surface was directly exposed to the gamma-rays.

**Supplementary Note 2**

**Effect of Bi_2_O_3_ concentration on the dosimetry response**

To investigate the effect of filler on the dosimetry response, as shown in Figure 1E, four samples containing 0, 20, 40, and 60 wt% Bi_2_O_3_ with dimensions of 2.3 cm×1 cm with thickness of 2 mm were fabricated based on the experimental procedure in the main manuscript (It is worth mentioning that in order to compare the dosimetry response, the dimensions of the dosimeters and copper electrode sheets in this case were made smaller than the B_60_ sample in the original manuscript). In the all samples, the weight percentage of GO was fixed at 0.1 wt%.

As can be seen from Figure 2E-a, effect of Bi_2_O_3_ nanoparticles wt% on the dosimetry response of the samples was investigated in different concentrations of 0, 20, 40, and 60 wt% at various dose rates. Results show that as the Bi_2_O_3_ wt% increases, the dosimeter response enhances subsequently. This trend is valid up to 40 wt%; but for 60 wt% sample, the dosimeter response decreases by 20-30% in comparison with the 40 wt% sample. To justify this phenomenon, it can be mentioned that due to the semi-crystalline nature of the polyethylene matrix in this study, the agglomeration occurs at the higher reinforcement loadings namely 60 wt% leading to decrease the dosimeter response subsequently. The dosimetry response of various Bi_2_O_3_ wt% at the fixed dose rate of 42.67 mGy/min is depicted in Figure 2E-b, in which exhibits a linear increase up to 40 wt%, then decreases 25% at 60 wt%.

| 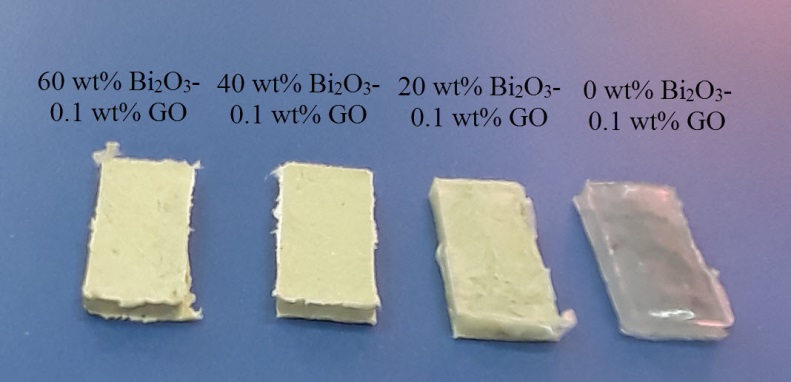 | 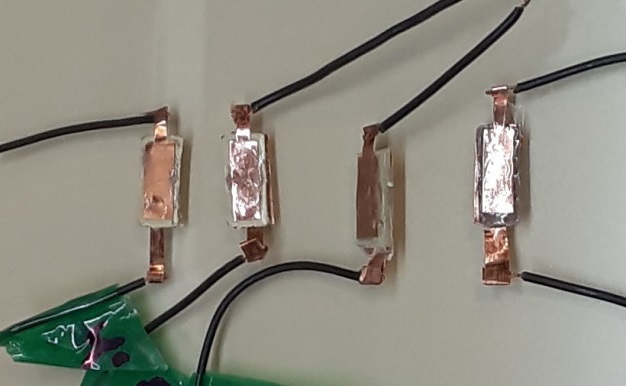 |
| --- | --- |

**Figure 1E.** Demonstration of the four samples prepared to study the effect of Bi_2_O_3_ additive on the dosimetry response.

|  |  |
| --- | --- |
| (a) | (b) |

**Figure 2E.** Effect of Bi_2_O_3_ wt% on the dosimetry response of the ternary nanocomposite in various loadings of 0, 20, 40, and 60 wt% with fixed value of 0.1 wt% GO, (a) at various dose rates, and (b) at dose rate of 42.67 mGy/min.

**Supplementary Note 3**

**Effect of GO concentration on the dosimetry response**

To investigate the effect of GO nanofillers on the dosimetry response, as shown in Figure 3E, different samples containing 0, 0.1, 0.5, 1, and 2 wt% GO with dimensions of 2.3 cm×1 cm and thickness of 2 mm were fabricated based on the experimental procedure in the main manuscript. As can be observed from Figure 3E, the color of the samples becomes darker with the addition of the GO wt%. In the all samples, the Bi_2_O_3_ wt% was kept fixed at 40 wt%, because this sample exhibited the maximum sensitivity among the other concentrations of the Bi_2_O_3_ inclusions in the previous section.

As can be seen from Figure 4E-a, effect of GO wt% on the dosimetry response of the samples was investigated for various concentrations of 0, 0.1, 0.5, 1, and 2 wt% (at the fixed value of 40 wt% for Bi_2_O_3_) at different dose rates. Results revealed that increasing the GO wt% led to enhancement of the dosimeter response significantly. To justify this behavior, it can be mentioned that according to the percolation theory [1-4], increasing the weight percentage of the carbonaceous nano-fillers in the polymer matrix leads to formation of more percolative networks in the sensitive volume of the dosimeter [5-8], in which prevents from the charge trapping in the nanocomposite during the irradiation. In fact, graphene oxide nanosheets act as the internal electrodes into the sensitive volume, in which by applying a suitable voltage, it prevents from the recombination of the radiation-induced-ions, thus will resulted in the improvement of the dosimeter response by adding the GO wt%. As can be seen from Figure 4E-b, a slight decrease in the response of the dosimeter in samples of 0.1, 0.5 wt% at the SSD=100 cm with dose rate of 25.5 mGy/min is probably due to the scavenger property of the graphene oxide, which is noticeable at low dose rates [9-11].

| 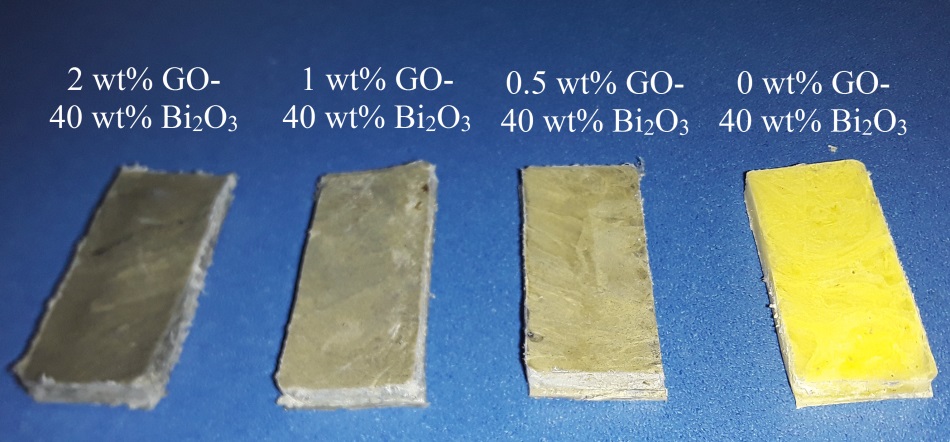 | 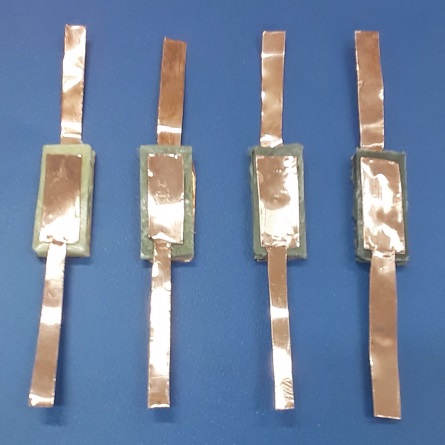 |
| --- | --- |

**Figure 3E.** Demonstration of four samples prepared to study the effect of GO additives on the dosimetry response.

|  |  |
| --- | --- |
| (a) | (b) |

**Figure 4E.** Effect of GO wt% on the dosimetry response of the ternary nanocomposite in various GO loadings of 0, 0.1, 0.5, 1, and 2 wt% with fixed value of 40 wt% Bi_2_O_3_, (a) at various dose rates, and (b) at dose rate of 39.84 mGy/min.

**Supplementary references**

[1] M. Sahimi, Applications of percolation theory, Taylor & Francis, London, 1994.

[2] C. Gao, S. Zhang, F. Wang, B. Wen, C. Han, Y. Ding, M. Yang, Graphene networks with low percolation threshold in ABS nanocomposites: selective localization and electrical and rheological properties, Applied Materials & Interfaces, 6 (2014) 12252-12260.

[3] J. Ma, J.T. Yeow, J.C. Chow, R.B. Barnett, Effect of percolation on electrical conductivity in a carbon nanotube-based film radiation sensor, Nanotechnology, 2008. NANO'08. 8th IEEE Conference on, IEEE, 2008, pp. 259-262.

[4] C.A. Martin, J.K.W. Sandler, M.S.P. Shaffer, M.K. Schwarz, W. Bauhofer, K. Schulte, A.H. Windle, Formation of percolating networks in multi-wall carbon-nanotube–epoxy composites, Composites Science and Technology, 64 (2004) 2309-2316.

[5] A. Veiskarami, D. Sardari, S. Malekie, F.B. Mofrad, S. Kashian, Computational prediction of electrical percolation threshold in polymer/graphene-based nanocomposites with finite element method, Journal of Polymer Engineering, (2022).

[6] S. Malekie, F. Ziaie, S. Feizi, A. Esmaeli, Dosimetry characteristics of HDPE-SWCNT nanocomposite for real time application, Nuclear Instruments and Methods in Physics Research Section A: Accelerators, Spectrometers, Detectors and Associated Equipment, 833 (2016) 127-133.

[7] S. Malekie, F. Ziaie, A. Esmaeli, Study on dosimetry characteristics of polymer–CNT nanocomposites: Effect of polymer matrix, Nuclear Instruments and Methods in Physics Research Section A: Accelerators, Spectrometers, Detectors and Associated Equipment, 816 (2016) 101-105.

[8] S. Malekie, F. Ziaie, Study on a novel dosimeter based on polyethylene–carbon nanotube composite, Nuclear Instruments and Methods in Physics Research Section A: Accelerators, Spectrometers, Detectors and Associated Equipment, 791 (2015) 1-5.

[9] H. Wang, K. Teng, C. Chen, X. Li, Z. Xu, L. Chen, H. Fu, L. Kuang, M. Ma, L. Zhao, Conductivity and electromagnetic interference shielding of graphene-based architectures using MWCNTs as free radical scavenger in gamma-irradiation, Materials Letters, 186 (2017) 78-81.

[10] W. Xia, H. Xue, J. Wang, T. Wang, L. Song, H. Guo, X. Fan, H. Gong, J. He, Functionlized graphene serving as free radical scavenger and corrosion protection in gamma-irradiated epoxy composites, Carbon, 101 (2016) 315-323.

[11] E. Kolanthai, S. Bose, K. Bhagyashree, S. Bhat, K. Asokan, D. Kanjilal, K. Chatterjee, Graphene scavenges free radicals to synergistically enhance structural properties in a gamma-irradiated polyethylene composite through enhanced interfacial interactions, Physical Chemistry Chemical Physics, 17 (2015) 22900-22910.
